# Do low-income coronary artery bypass surgery patients have equal opportunity to access excellent quality of care and enjoy good outcome in Taiwan?

**DOI:** 10.1186/s12939-014-0064-8

**Published:** 2014-09-10

**Authors:** Tsung-Hsien Yu, Yu-Chang Hou, Kuo-Piao Chung

**Affiliations:** Institute of Healthcare Policy and Management, National Taiwan University, Taipei, Taiwan; Department of Chinese Medicine, Tao-Yuan General Hospital, Ministry of Health and Welfare, Taoyuan, Taiwan; Department of Bioscience Technology, Chuan-Yuan Christian University, Taoyuan, Taiwan

**Keywords:** Quality of care, Health inequity, Patterns of care, CABG

## Abstract

**Background:**

Equity is an important issue in the healthcare research field. Many studies have focused on the relationship between patient characteristics and outcomes of care. These studies, however, have seldom examined whether patients’ characteristics affected their access to quality healthcare, which further affected the care outcome. The purposes of this study were to determine whether low-income coronary artery bypass surgery (CABG) patients receive healthcare services with poorer quality, and if such differences in treatment result in different outcomes.

**Methods:**

A retrospective multilevel study design was conducted using claims data from Taiwan’s universal health insurance scheme for 2005-2008. Patients who underwent their CABG surgery between 2006 and 2008 were included in this study. CABG patients who were under 18 years of age or had unknown gender or insured classifications were excluded. Hospital and surgeon’s performance indicators in the previous one year were used to evaluate the level of quality via k-means clustering algorithm. Baron and Kenny’s procedures for mediation effect were conducted to explore the relationship among patient’s income, quality of CABG care, and inpatient mortality.

**Results:**

A total of 10,320 patients were included in the study. The results showed that 5.65% of the low-income patients received excellent quality of care, which was lower than that of patients not in the low-income group (5.65% vs.11.48%). The mortality rate of low-income patients (12.10%) was also higher than patients not in the low-income group (5.25%). Also, the mortality of patients who received excellent care was half as low as patients receiving non-excellent care (2.63% vs. 5.68%). Finally, after the procedure of mediation effect testing, the results showed that the relationship between patient income level and CABG mortality was partially mediated by patterns of quality of care.

**Conclusions:**

The results of the current study implied that worse outcome in low-income CABG patients might be associated with poorer quality of received services. Health authorities should pay attention to this issue, and propose appropriate solutions.

## Introduction

Health inequality is an important issue all around the world. Many countries have been trying to increase the accessibility to health care resources and provide better healthcare services for their people. Studies regarding the health inequality issue have found influences of racial/ethnic [1-3], socioeconomic [4,5] and geographic [6-8] factors on health status (e.g., disease status [5,9] or mortality [10,11]), healthcare resources utilization [8,12,13], and outcomes (e.g., postoperative mortality rates [14]). Most of the results from these studies have pointed to positive relationships between the indicators of social status and various health outcomes [15-17]. In general, higher socioeconomic status has been linked to better healthcare outcomes [18-22]; however, previous studies have failed to consider the patterns in quality of care delivered. Many studies have shown that quality of care is an important factor to improve the outcome of healthcare; therefore, the role of quality of care should be considered when studying the health inequality issue.

In Taiwan, several occupation-based social insurance schemes originally covered healthcare services beginning 1950, and 56% of the entire population were under these insurance schemes. However, these health insurance programs were mainly available to people under an employee-employer relationship. Children under 14 years of age, elder people (over 65 years old), the unemployed, and a great proportion of women were not protected by any social insurance programs. To enable equal accessibility to healthcare, the government of Taiwan consolidated all of its social insurance programs and launched the National Health Insurance (NHI) program on March 1, 1995 to cover the entire civilian population [23]. Taiwan’s NHI program is a single-payer compulsory social insurance plan which centralizes the disbursement of healthcare funds, and the population coverage has reached 99%. The insured can be categorized into six classifications according to their occupation. Classifications 1 to 4 are for people who are under employment (e.g. civil servants, employees, employers, farmers/fishermen, military personnel), the fifth classification is for households below the poverty line, and the sixth is for veterans and other individuals. Dependent family members should enroll through their closest blood relative’s (e.g. parent, spouse and children) insurance registration organization.

Even with the universal health insurance program, several domestic studies have shown that inequality in health services has continued to exist. Chen et al. conducted a study in Taiwan and found that the gaps in income and education levels between southern and northern people have become wider during the past 20 years [24]. A study by Wen et al. revealed that although life expectancy has improved for lower-income people since the introduction of national health insurance, the magnitude of the reduced disparity has been small compared with the size of the remaining gaps between economic groups [25].

Coronary artery *bypass surgery* (CABG) is a high-risk surgery with mortality of around 5% [26]. Many quality indicator projects (e.g. International Quality Indicator Project) have adopted mortality as an indicator to monitor the quality of CABG. Therefore, the current study takes CABG as an example to examine whether the mortality rate differs by patients’ income level; if so, we further investigate whether the relationship between patient income level and mortality is mediated by quality of care.

## Methods

### Study design

**Figure 1:**
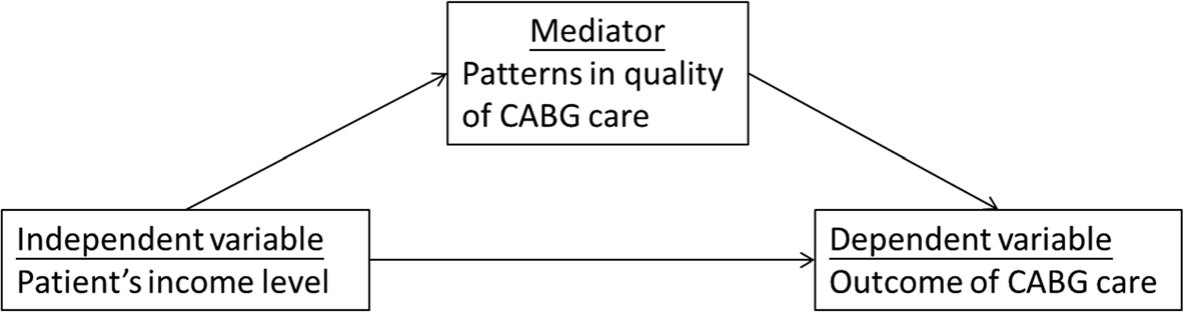
**Conceptual framework of a mediator effect.**

This retrospective and cross-sectional study adopted a multilevel design to examine the relationships between patient income level, patterns of quality of healthcare, and treatment outcomes among CABG patients after adjusting by patient-, surgeon-, and hospital-level covariates. The conceptual framework of this study is demonstrated in Figure 1.

### Database

We used data from the Taiwan National Health Insurance Research Database (NHIRD) from between 2005 and 2008. The NHIRD, published by the Taiwan National Health Research Institute, includes all the original claims data and registration files for beneficiaries enrolled under the NHI program. This database covers the 23 million enrollees in the NHI program (approximately 98% of Taiwan’s population). The records system provides de-identified, secondary patient-level demographic, administrative information and discharge status on every case, and this database can be accessed by the public for research purposes [27]. The protocol for this study was approved by the Institutional Review Board of the National Taiwan University Hospital (protocol #201312115 W).

### Study population and Exclusion criteria

**Figure 2:**
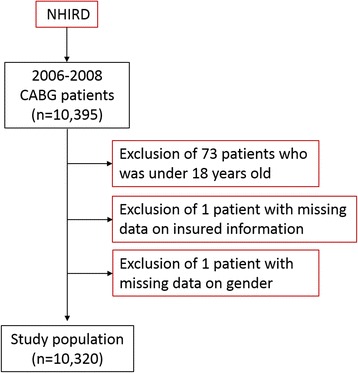
**Flow recruitment chart of subjects from the National Health Insurance Research Database (NHIRD), 2006 to 2008, in Taiwan.**

We restricted our analysis to hospitalization records in which patients had a procedure code indicating a CABG (*International Classification of Diseases, 9th ed. Clinical Modification* [ICD-9-CM] procedure codes 35.1x–36.2x) [14] between 2006 and 2008. We excluded patients under the age of 18 years (N = 83) to restrict our evaluation to an adult population. Hospitalization records with missing data for insured classification (n = 1) or gender (n = 1) were excluded (see Figure 2).

### Definition of variables

#### Dependent variable: Inpatient mortality

Cases of inpatient mortality were drawn from the claims data. These included all cases of in-hospital death following CABG, or of critical cases discharged against advice (AAD). People in Taiwan, especially the elderly, prefer to die at home. Therefore, patients who had critical AAD record in their discharge code will be also identified as mortality cases.

#### Independent variable: Income level

Patients’ insurance identification records were used to distinguish patients in the low-income group from those who were not. As mentioned above, households below the poverty line belonged to classification 5. We used this information in NHIRD as a criterion to identify the income level.

#### Mediator variable: Patterns of quality of care

Since Donabedian proposed a quality of care framework that comprised three dimensions (structure, process and outcome) [28], researchers have had a more comprehensive frame for evaluating the quality of care. Our study calculated the risk-adjusted inpatient mortality rates (outcome), infection rates (outcome), and service volumes (structure, it represented the experience or skill of a healthcare provider) of each hospital and for each surgeon in the previous one year before each CABG surgery to evaluate the quality of CABG. The procedure used in determining risk adjustment for the inpatient mortality and infection rates followed the recommendations of Daley et al. [29]. Data on patient gender, age, Charlson/Romano Comorbidity Index (CCI) and number of vessels obstructed were incorporated for risk adjustment.

Previous studies usually categorized quality in a subjective manner to explore the relationship between quality and outcomes of care [30,31]. However, some studies indicated the results might be influenced by the categorization methods [32,33]. To avoid subjectivity, and to allow us to consider these three quality indicators simultaneously, this study adopted k-means clustering algorithm to classify the quality of hospitals and surgeons. K-means clustering algorithm was based on cluster analysis. It is a kind of data mining approach, and is also one of the most used methods for partitioning clusters. The aim of this method was to identify representative data points, also called cluster centers, prototypes, or code words, from large volumes of high-dimensional data [34]. Kuo and his colleagues applied this approach in categorizing the provider’s service volume to explore the relationship among volume, mortality, and recurrence in breast cancer [35].

The K-means clustering algorithm was employed by SPSS version 16 software. Risk-adjusted inpatient mortality rates, infection rates, and service volumes were used as parameters, and inpatient mortality was used as the outcome variable. Surgeons and hospitals were assigned to “good performance” and “non-good performance” groups according to their distance to cluster centers. Patients who went to a “good performance” hospital and received healthcare from a “good performance” surgeon were included in the “excellent care” group, and the other patients were classified in the “non-excellent care” group.

### Control variables

In addition to three important patient-level variables we mentioned above, this study also collected other patient-, surgeon-, and hospital-level data. First, patient-level variables included age, gender, Charlson/Romano Comorbidity Index, and number of obstructed vessels (as a proxy indicator of duration of operation [36]) that were involved in the surgical operation. Second, surgeon-level variables included age and gender. Third, hospital-level variables included hospital ownership and accreditation status.

### Statistical analysis

All statistical analyses were performed using *SAS* (version 9.2, SAS Institute Inc., Cary, NC, USA). In statistical testing, a two-sided *p* value ≤ 0.05 was considered statistically significant. The distributional properties of continuous variables were expressed by mean ± standard deviation (SD), and the categorical variables were presented by frequency and percentage. In univariate analysis, potential predictors of CABG inpatient mortality and income status were examined using the chi-square test or the two-sample *t-*test as appropriate.

To account for correlations of information within the healthcare provider, multivariate analysis was conducted by fitting multilevel or mixed-effects logistic regression models to each patient’s data, and then estimating the effects of hospital- and surgeon-level predictors on the probability of CABG inpatient mortality.

To ensure the analysis quality, basic model-fitting techniques for (a) variable selection, (b) goodness-of-fit (GOF) assessment, and (c) regression diagnostics were used in our regression analyses. Specifically, the stepwise variable selection procedure (with iterations between the forward and backward steps) was applied to obtain the best candidate final regression model. The significance levels for entry (SLE) and for stay (SLS) were set as 0.20 or larger for the sake of conservativeness. Any discrepancies between the results of univariate analysis and multivariate analysis were probably due to the confounding effects of the uncontrolled covariates in univariate analysis. The GOF measures (including the percentage of concordant pairs, the estimated area under the receiver operating characteristic curve and the adjusted generalized *R*^2^) were examined with the Hosmer-Lemeshow GOF test to assess the GOF of the fitted multilevel or mixed-effects logistic regression model. The statistical tools for regression diagnostics such as residual analysis, detection of influential cases and checks for multicollinearity were applied to discover any model or data problems.

In addition, we combined Baron and Kenny’s mediation effect testing procedure [37] with the recommendations given by Mathieu et al. [38] to examine the relationships among income status, patterns of quality of care, and inpatient mortality. There are three steps to test the linkages of the mediational model. To establish mediation, the following conditions must hold. First, the independent variable must be shown to affect the dependent variable; second, the independent variable must affect the mediator; and third, the mediator must affect the dependent variable in the third equation. If these conditions all hold in the predicted direction, then the effect of the independent variable on the dependent variable must be less in the third step than in the first step (partial mediator). Perfect mediation holds if the independent variable has no effect when the mediator is controlled. Finally, Sobel’s test was used for the significance of the mediation test [39].

## Results

There were 10,320 CABG surgeries performed by 322 surgeons in 60 hospitals during 2006-2008 in Taiwan, and 124 (1.20%) of them were for low-income patients. Around two thirds of the observed patients received their operations in medical centers. With respect to hospital ownership, more than one-third of the patients went to a public hospital, 10,027(97.22%) patients received care from a male surgeon, with the mean age of these surgeons being 44 years, around one-third of patients were female, and the mean age of their surgeons was 65 years. Around 60% of patients had more than two vessels being obstructed, and 550 patients (5.33%) died after the surgery. The results of k-means clustering algorithm showed that 5,396 (52.29%) patients were treated by “good performance” surgeons, and 2,326 (22.57%) patients were treated in “good performance” hospitals. A total of 1,178 (11.41%) patients received excellent care (good performance hospital and good performance surgeon) during their hospitalization.

In terms of hospital selection, the data showed no significant differences in hospital level or ownership type chosen by low-income or non-low-income groups. Considering hospital performance, hospitals selected by low-income patients had lower service volumes (124.90 vs. 147.83) and worse risk-adjusted inpatient mortality rates (7.48% vs. 5.39%). However, these hospitals had risk-adjusted infection rates similar to those of hospitals selected by non-low-income patients. Low-income patients tended to receive care from younger surgeons (42.77 vs. 44.48). Regarding surgeon performance, surgeons who served low-income patients had lower service volumes (44.40 vs. 50.46) and worse risk-adjusted inpatient mortality rates (6.40% vs. 4.88%). The surgeon-level risk-adjusted infection rates also did not differ significantly between surgeons serving the two patient groups. The study results also showed that low-income patients were younger, and were more likely to have comorbidity issues. Gender and number of vessels obstructed, however, were not different between the two patient groups. Inpatient mortality rate was higher in low-income patients (12.10% vs. 5.25%). The results also revealed that the percentage of low-income patients who received excellent quality of care was lower than non-low-income patients (Table 1). With respect to patterns of quality of care, hospital accreditation status and three quality indicators, surgeon’s gender, patient’s age, and number of vessels obstructed differed significantly between the two patterns of quality of care, so that inpatient mortality of the excellent group was twice as low as the non-excellent group (2.63% vs. 5.68%).

**Table 1:** **Descriptive analysis: stratified by income status and patterns of quality of care**

		**Income level**			**Patterns of care**		
	**All**	**Non-poor**	**Poor**	***p*** **-value**	**Not excellent**	**Excellent**	***p*** **-value**
	**(** ***n*** **=10,320)**	**(** ***n*** **=10196)**	**(** ***n*** **=124)**		**(** ***n*** **=9,142)**	**(** ***n*** **=1,178)**	
**Hospital-level**							
Accreditation status, *n* (%)				0.6392^†^			<0.0001^†^
Medical center	6955(67.39)	6869(67.37)	86(69.35)		6395(69.95)	560(47.54)	
Not medical center	3365(32.61)	3327(32.63)	38(30.65)		2747(30.05)	618(52.46)	
Ownership, *n* (%)				0.5134^†^			0.4790^†^
Public hospital	3706(35.91)	3658 (35.88)	48 (38.71)		3272(35.79)	434(36.84)	
Not public hospital	6614(64.09)	6538 (64.12)	76 (61.29)		5870(64.21)	744(63.16)	
Hospital service volume, mean (S.D.)	147.56(92.45)	147.83(92.53)	124.90(82.40)	0.0060^‡^	151.12(90.68)	119.89(101.03)	<0.0001^‡^
Hospital risk-adjusted infection rate (%), mean (S.D.)	1.31(1.83)	1.31(1.82)	1.43(2.13)	0.5238^‡^	1.40(1.86)	0.57(1.25)	<0.0001^‡^
Hospital risk-adjusted mortality rate (%), mean (S.D.)	5.41%(5.53)	5.39(5.40)	7.48(12.10)	0.0504^‡^	5.79(5.61)	2.45(3.68)	<0.0001^‡^
**Surgeon-level**							
Surgeon’s gender, *n* (%)				0.1783^†^			0.0003^†^
Female	287(2.78)	286(2.81)	1 (0.81)		235(2.57)	52(4.41)	
Male	10027(97.22)	9904(97.19)	123 (99.19)		8901(97.43)	1126(95.59)	
Surgeon’s age, mean (S.D.)	44.46(7.65)	44.48(7.65)	42.77(6.82)	0.0134^‡^	44.48(7.63)	44.29(7.79)	0.4244^‡^
Surgeon service volume, mean (S.D.)	50.38(34.65)	50.46(34.66)	44.40(32.94)	0.0439^‡^	50.12(33.46)	52.46(42.70)	0.0285^‡^
Surgeon risk-adjusted infection rate (%), mean (S.D.)	1.28(3.18)	1.28(3.18)	1.27(2.76)	0.9568^‡^	1.39(3.31)	0.50(1.63)	<0.0001^‡^
Surgeon risk-adjusted mortality rate (%), mean (S.D.)	4.89(7.31)	4.88 (7.24)	6.40(11.71)	0.0236^‡^	5.29(7.53)	1.86(4.30)	<0.0001^‡^
**Patient-level**							
Age, mean (S.D.)	65.35(10.95)	65.40(10.93)	61.38(11.74)	<0.0001^‡^	65.44(10.96)	64.65(10.84)	0.0187^‡^
Gender, *n* (%)				0.1289^†^			0.1504^†^
Female	2424(23.49)	2402(23.56)	22(17.74)		2167(23.70)	257(21.82)	
Male	7896(76.51)	7794(76.44)	102(82.26)		6975(76.30)	921(78.18)	
CCI, *n* (%)				<0.0001^†^			0.6394^†^
<=1	5453(52.84)	5412(53.08)	41(33.06)		4823(52.76)	630(53.48)	
2+	4867(47.16)	4784(46.92)	83(66.94)		4319(47.24)	548(46.52)	
Number of vessels obstructed, *n* (%)				0.5094^†^			<0.0001^†^
1	4278(41.45)	4223(41.42)	55(44.35)		3634(39.75)	644(54.67)	
2+	6042(58.55)	5973(58.58)	69(55.65)		5508(60.25)	534(45.33)	
Patterns of quality of care							
Not excellent, *n* (%)	9142(88.59)	9025(88.52)	117(94.35)	0.0421^†^			
Excellent, *n* (%)	1178(11.41)	1171(11.48)	7(5.65)				
Inpatient mortality, *n* (%)	550(5.33)	535 (5.25)	15(12.10)	0.0007^†^	519(5.68)	31(2.63)	<0.0001^†^

^†^ *χ* ^2^ test ^‡^ *t*-test.

Finally, the study included all of the controlled variables in the multilevel logistic regression model. After stepwise selection, patient gender, age, CCI, number of vessels obstructed, and hospital *accreditation status* remained in the model (see Table 2). Table 3 demonstrated the results of multilevel mediation effect examination. Model 1 served to verify any linkage between income level and postoperative inpatient mortality. After controlling for other covariates, the results suggested that non-low-income patients were associated with a lower postoperative inpatient mortality risk (OR = 0.353, 95%; CI = 0.193 ~ 0.644) than the low-income patient group. Model 2 shows the relationship between income level and patterns of quality of care. The results from this model indicated that low-income patients were less likely to receive high quality healthcare (OR = 2.291, 95%; CI = 1.033 ~ 5.078/β = 0.8828, standard error = 0.3985). Model 3 tested whether a mediation effect from patterns of quality of care existed within the relationship between income level and postoperative inpatient mortality. The results indicated that when income level and patterns of quality of care were placed in the model, the non-excellent group had higher mortality risk (OR = 2.632, 95%; CI = 1.737 ~ 3.960/β = 0.9642, standard error = 0.2080) than the excellent group, after adjusting for income level and other covariates. Furthermore, the effect of income level on inpatient mortality decreased from 0.353 to 0.369 (OR = 1 means no effect). The result of Sobel’s test showed significant mediation effect.

**Table 2:** **Results of stepwise selection**

	**Full model**			**Parsimonious model**		
	***β***	***S.E.***	**p-value**	***β***	***S.E.***	**p-value**
Accreditation status (ref = medical center)	0.1279	0.0495	0.0098	0.1378	0.0461	0.0028
Ownership (ref = public)	0.0236	0.0506	0.6409			
Surgeon’s gender (ref = male)	0.0376	0.1247	0.7630			
Surgeon’s age	–0.0474	0.00686	<.0001	–0.0474	0.00688	<.0001
Patient’s age	0.0531	0.00482	<.0001	0.0529	0.00481	<.0001
Patient’s gender (ref = male)	0.1220	0.0484	0.0117			
CCI (ref = 2+)	0.3561	0.0475	<.0001	0.3549	0.0473	<.0001
Number of vessels obstructed (ref = 2+)	–0.2322	0.0463	<.0001	–0.2319	0.0463	<.0001

**Table 3:** **Results of multilevel analysis: mediation effect examination**

	**Model 1** ***(y=mortality)***			**Model 2** ***(y=patterns of QOC)***			**Model 3** ***(y=mortality)***		
	**Odds ratio**	**95% C.I.**		**Odds ratio**	**95% C.I.**		**Odds ratio**	**95% C.I.**	
		**LCL**	**UCL**		**LCL**	**UCL**		**LCL**	**UCL**
Fixed-effects									
**Hospital-level**									
Accreditation status (ref. = Medical center)	1.322	0.904	1.932	2.401***	1.530	3.767	1.410	0.971	2.048
**Surgeon-level**									
Surgeon’s age	0.954***	0.933	0.976	1.005	0.981	1.029	0.954***	0.933	0.975
**Patient level**									
Income(ref. = low income)	0.353***	0.193	0.644	2.291*	1.033	5.078	0.369**	0.202	0.673
Patterns of QOC *(ref.=excellent)*							2.623***	1.737	3.960
Age	1.055***	1.045	1.066	0.991**	0.985	0.998	1.054***	1.044	1.065
Gender (ref. = Male)	1.292**	1.058	1.579	0.848*	0.719	1.000	1.280*	1.047	1.565
Number of vessels obstructed (ref. = >2+ vessels)	2.029***	1.641	2.509	1.670***	1.425	1.957	2.100***	1.699	2.595
Comorbidity index (ref. = 2+)	0.637***	0.526	0.770	1.029	0.896	1.180	0.634***	0.524	0.767
Random effects	Variance	Std.err.		Variance	Std.err.		Variance	Std.err.	
Intercept: surgeon (hospital)	0.6100	0.2796		1.1590	0.2978		0.5419	0.2619	
Intercept: surgeon	0.0234	0.2602		0.1473	0.2226		0.02730	0.2428	

*p<0.05 **p<0.01 ***p<0.001.

QOC: quality of care; C.I.: confidence interval; LCL: Lower confidence limit; UCL: Upper confidence limit.

$$ \left(\mathrm{t}=\left(0.8828 \times 0.9642\right)/\sqrt{(0.8828)^2{(0.2080)}^2+{(0.9642)}^2{(0.3985)}^2}=2.00>1.96\right) $$

In that case, according to Baron and Kenny’s recommendation [37], the relationship between a patient’s income level and the CABG inpatient mortality was partially mediated by patterns of quality of care. However, as the percentage of low-income patients was very low, we randomly selected 496 patients from the non-low-income group for further analysis to confirm the results. The results supported previous findings (Table 4).

**Table 4:** **Summary results of multilevel analysis: mediation effect examination (random sampling 1:4 case-control)**

	**Model 1** ***(y=mortality)***			**Model 2** ***(y=patterns of QOC)***			**Model 3** ***(y=mortality)***		
	**Odds ratio**	**95% C.I.**		**Odds ratio**	**95% C.I.**		**Odds ratio**	**95% C.I.**	
		**LCL**	**UCL**		**LCL**	**UCL**		**LCL**	**UCL**
Fixed-effects									
Income (ref. = low income)	0.392**	0.176	0.876	2.562*	1.099	5.973	0.420*	0.190	0.933
Patterns of QOC *(ref. = excellent)*							6.277**	1.147	34.354

*p<0.05 **p<0.01 ***p<0.001.

QOC: quality of care; C.I.: confidence interval; LCL: Lower confidence limit; UCL: Upper confidence limit.

Adjusted for surgeon’s age, cci, and Number of vessels obstructed.

## Discussion

Better equity in healthcare service, with reduction of any gaps in access in terms of gender, income level, socioeconomic status or residential area differences, has long been an important goal of global healthcare reform. The results of this study reveal that even with the National Health Insurance program, people in Taiwan have different levels of access to quality healthcare. Populations with low income are less likely to approach high-quality healthcare providers, and this tendency leads to poorer treatment outcomes for these individuals.

The premium of Taiwan NHI is based on insured monthly salary [40,41]. Existing studies that have used the NHIRD to discuss the health inequality issues on income level in Taiwan usually employed monthly insurance premiums as the basis for classification. However, some employers do not buy insurance for their workers based on their real salary levels. Dependent, retired, or unemployed populations can freely attach their insurance to their closest relatives. As all of these factors could cause bias when patient income level was categorized according to their monthly insurance premium, our study adopted the insured classifications to differentiate low-income individuals from the others. Households with income below the poverty line were recognized as low-income households by the government. Therefore, our identification of low-income patients should be reliable.

Furthermore, most of the previous studies, especially those studying the service volume-outcome relationship, used data of the previous one year before study period to classify the provider’s quality. Our study data covered three years, and therefore hospital and surgeon quality might change over time. If so, hospital and surgeon quality of the previous one year before study period might be different with the quality in the second or third year of the study period. Therefore, we selected the risk-adjusted inpatient mortality rates, risk-adjusted infection rates, and service volume of each hospital and for each surgeon in the previous one year before each CABG surgery, rather than before the study period.

In addition to the main finding, the study results suggested another two points that are worth discussing:

1. Categorization of patterns of quality of care
  Quality of care has long been one of the three major issues in the healthcare delivery system. For years, medical researchers have made great efforts to develop methods for evaluating the quality of care. At first, such research relied mainly on peer-reviews [42]. Donabedian’s quality of care framework [28] is the most common to evaluate the quality of care. Many quality indicator projects applied his framework to develop quality indicator and to measure healthcare quality. Nevertheless, too many indicators can make interpretation difficult. Therefore, a transformation algorithm is required to understand the meaning of quality indicators in a simple manner. Currently, the most popular method is based on composite scores, which can be determined through several approaches: (a) all or none, (b) 70% standard, (c) overall percentage, (d) indicator average, or (e) patient average [43,44]. These five calculation approaches are all associated with varying advantages and disadvantages, and thus can be fitted to different purposes [44]. Also, these methods all involve a certain degree of subjective judgment, so some researchers have begun to use the latent approach to interpret quality indicators [45]. The latent approach is based on statistical methods and gives weights to different indicators to obtain a universal appraisal. The k-means clustering method adopted in this study assists in categorization based on statistics. To avoid the problems mentioned above and yet capture the universal quality of care, we used CABG service volumes, risk-adjusted infection rates, and inpatient mortality rates to represent the structure and outcome dimensions respectively. We then applied k-means clustering to help in categorizing quality of care.
2. Relationships among income status, patterns of quality of care, and healthcare outcomes
  Previous studies regarding the inequality of CABG outcomes have primarily emphasized differences in gender [46,47] and ethnicity [3,48]. Few studies have discussed the influence of healthcare providers [49]. Although many studies have supported a relationship between high socioeconomic status and better healthcare outcomes, no study has seriously explored the reasons for this phenomenon. Our study provided new insight that the relationship between patient income and healthcare outcome was mediated by patterns of quality of care. However, according to Baron and Kenny’s mediator effect testing procedure, quality of care was not a perfect mediator in the current study. The representativeness of these three quality indicators is an issue worthy for further discussion. As we mentioned above, the three indicators we used in this study were represented as structure and outcome dimension indicators. However, can they represent the overall quality of CABG care? These indicators were the most common for CABG quality indicators, and many agencies, domestic and international organizations (e.g. *Agency for Healthcare Research and Quality of United State*, Organization for Economic Co-operation and Development, International Quality Indicator Project) have adopted these indicators for different purposes. We believe that there are other indicators that should be included (e.g. CABG guideline adherence rate), but these indicators are not available in the Taiwan NHIRD. It might explain why the relationship between income level and inpatient mortality was only partially mediated by patterns of quality of care.

### Policy implication

Although the implementation of Taiwan’s National Health Insurance has reduced the financial barriers to health care, our findings showed that health disparity still existed in CABG patients. This phenomenon might be impacted by patterns of quality of care. Low-income CABG patients, compared to non-low-income patients, had fewer opportunities to assess better quality of care and had worse mortality. The health authority should improve this situation in addition to continuous improvement of the quality of CABG care, and reduce the variance of care among providers. Furthermore, some studies indicated that low-income people had worse health literacy [50]. Enhancing patient’s information in health-seeking might also reduce disparity. Public disclosure or report card is the common approach to provide information to patients for surgeon or hospital selection.

### Limitations

Unlike previous studies, the current study not only took patient income level to examine its relationship with outcome of care, but also took the level of quality of care to examine the relationship among these three variables after adjusting for other patient’s and healthcare provider’s characteristics. Furthermore, the study managed the nested data issue via a multilevel study design. However, even with these advantages, the study was still subject to several limitations as described below:

1. Mortality cases identification. The study adopted postoperative inpatient mortality as the dependent variable, although the indicator of 30 days mortality after CABG is more commonly used around the world. As the claims data used in this study could not be linked to the Taiwan National Death Certificate Registry, only data concerning inpatient deaths could be used. These data tend to be incomplete, as it is a custom in Taiwan that many people, especially the elderly, prefer to die at home. That was also the reason we identified the critical AAD patients as mortality cases.
2. Patients’ income status as revealed in the data, can be different from the reality. We could not obtain the tax information of each patient, and therefore could only rely on the insurance information (insurance identification and type) to identify low-income individuals. As the health insurance program in Taiwan calculates each person’s premium based on his/her monthly salary, those in the non-low-income group were not further divided accurately. Considering that our study concerned the disparity of healthcare outcomes between low-income and non-low-income groups, this limitation did not affect the study results.
3. Un-measureable variables. Although the study used the number of vessels obstructed and the comorbidity index as proxy indicators for disease severity and health status, other variables such as nutrition status (which can also affect inpatient mortality) were not collected. Moreover, our study could only measure the relationship between patient income level and patterns of quality of care, but not the causality for this relationship.

## Conclusions

Health is a natural right, and every government should provide sufficient and quality healthcare services for their people. To eliminate health inequity is a challenge that healthcare delivery systems of each country should deal with. This study shows that patients with low income are less likely to approach the better-performing healthcare providers, and this tendency indirectly affects their treatment outcomes. People in Taiwan enjoy greater equity in healthcare service, but policymakers might still need to develop strategies to ensure better equity in access to quality healthcare among people of different income level groups.
